# Progressive Spastic Paraparesis Caused by Thoracic Spinal Epidural Lipomatosis: A Case Report and Systematic Review

**DOI:** 10.3390/medsci14050559

**Published:** 2026-09-10

**Authors:** Wojciech S. Gaweł, Justyna M. Fercho, Olga Kałużna, Zuzanna Krasula, Mateusz Tyburski, Julia Nakoneczna, Remigiusz Kopeć, Jacek Szypenbejl, Jakub Soboń, Marcin Birski, Jacek Furtak, Mariusz Siemiński

**Affiliations:** 1 Scientific Circle of Neurotraumatology, Department of Emergency Medicine, Medical University of Gdańsk, 80-952 Gdansk, Poland; 2 Department of Emergency Medicine, Medical University of Gdańsk, 80-214 Gdansk, Poland; 3 Neurosurgery Department, 10th Military Research Hospital and PolyClinic SPZOZ in Bydgoszcz, 85-681 Bydgoszcz, Poland; 4 Faculty of Medicine, Bydgoszcz University of Science and Technology, 85-796 Bydgoszcz, Poland

**Keywords:** spinal epidural lipomatosis, SEL, MRI, case report, systematic review, spastic paraperesis, bilateral laminectomy

## Abstract

**Background:** Spinal epidural lipomatosis (SEL) is a rare disorder characterized by excessive epidural fat accumulation. Diagnosis may be challenging because imaging findings are frequently overlooked, and evidence-based management guidelines, particularly thoracic disease, remain limited. **Case report:** We present a case report of a 54-year-old male patient, who suffered from lower limb spastic paresis and associated muscle spasms (Nurick grade V). After a difficult diagnostic process, MRI of the thoracic spine confirmed dorsal/posterior spinal epidural lipomatosis (SEL; Manjila grade IIb-α) extending from Th2 to Th9, with moderate thecal sac compression. A small Th6–Th7 intervertebral disc prolapse was also present, without evidence of scoliosis, syrinx, spinal cord edema, or myelomalacia. Following bilateral thoracic decompression and complete resection of the SEL tissue, the patient’s neurological function improved markedly, from Nurick grade V preoperatively to Nurick grade II at 11 months of follow-up. Consequently, bilateral laminectomy of the thoracic region was performed with total resection of the SEL tissue. Following surgery, the patient’s neurological symptoms improved markedly (Nurick grade II; 11 months postoperatively), and histopathological examination confirmed the diagnosis of spinal epidural lipomatosis. **Methods:** We performed a systematic review of case reports of SEL occurring, or co-occurring in the thoracic region based on the literature identified on PubMed, Scopus and Web of Science databases, after using search terms: “(thoracic) AND (“spinal” OR “epidural”) AND (lipomatosis)” and filters: [English], [humans] and [year 2020–2026], with the accordance to the PRISMA guidelines. **Results:** We identified 21 cases of thoracic SEL from 19 articles. In 12 cases the patients were male, in 9 female. Reported etiological associations included type 1 diabetes mellitus, exogenous corticosteroid exposure, obesity/metabolic syndrome, Cushing syndrome, and CLOVES syndrome. Given the small, case-report-based sample, these findings should be considered reported associations rather than estimates of prevalence or independent risk factors. Surgical treatment of thoracic SEL can be accomplished by various techniques such as laminectomy (continuous or skip), laminoplasty, or minimally invasive procedures—including endoscopic spine surgery. **Conclusions:** Large-volume, multicenter studies with long-term clinico-radiological follow-up would be necessary to understand the natural history and treatment outcomes, and to form evidence-based guidelines for interventions. Our case report and findings highlight that thoracic SEL should be included in the differential diagnosis of unexplained compressive myelopathy, even when imaging studies are inconclusive. Rapid identification or possibly reversible causes of myelopathy and initiation of treatment can significantly relieve symptoms, so it is important to remember this disease in the diagnostic process.

## 1. Introduction

Spinal Epidural Lipomatosis (SEL) is a rare cause of spinal cord disorders [1], resulting from excessive accumulation of epidural adipose tissue [2]. SEL is most commonly associated with obesity, chronic corticosteroid therapy, and endogenous endocrinopathies such as Cushing syndrome—all of which promote epidural fat deposition. Symptoms usually develop once epidural adipose tissue causes compression of the thecal sac or spinal cord [3].

SEL detection is based on radiological imaging, with MRI being preferred over CT, however, both modalities present different approaches to the disorder. MRI remains the imaging modality of choice due to its superior soft tissue visualization, yet CT may also demonstrate epidural fat accumulation, which can be helpful in the diagnostic process [4]. Borre at al 2003 [5] defined detection and grading criteria for SEL based on radiometric indices (DuS/EF and EF/SC) from MRI measurements, however the original metrics were validated on patients with SEL of lumbo-sacral region. No widely accepted criteria exist for SEL of thoracic spine. Definitive confirmation of SEL is often done by performing a histopathological analysis of the surgical specimen—to exclude malignant growth or other diseases. Microscopic investigation of SEL tissue reveals proliferation of mature adipocytes, often with Y chromosome loss [6].

Management of the disease can be broadly divided into conservative and surgical. Weight reduction remains an important component of conservative management in patients with obesity-associated SEL. In selected patients with severe obesity, bariatric surgery may be considered as part of a comprehensive weight-management strategy, although evidence specifically addressing its effect on SEL remains limited. Pharmacological weight-loss therapies, including GLP-1 receptor agonists, may represent additional options in selected patients [7]. Surgical treatment aims to relieve spinal cord or thecal sac compression and may include laminectomy, laminoplasty, or minimally invasive procedures, including endoscopic spine surgery, with or without direct resection of the epidural adipose tissue. Conservative treatments are often used as a first line treatment before the surgery and as an additional therapy after the operation, to prevent disease reoccurrence [1].

In our article, we report a case of a 54-year-old male patient with thoracic Th2–Th9 SEL, co-occurring with Th6–Th7 intervertebral disc herniation, causing spastic paralysis of the lower limbs. We also performed a literature review of relevant literature, published in the years 2020–2026, to analyze rate of occurrence of thoracic SEL as a cause of myelopathy and showcase its possible symptoms, radiological findings and treatment options, as well as to highlight the difficulties in diagnosing the disease.

## 2. Case Report

A 54-year-old male Caucasian patient, with BMI of 36, was admitted with a history of spastic paraparesis and associated muscle spasms worsening over the previous 30 months. Episodes of symptom exacerbation could be provoked by long distance walking, stress and cold exposure. These episodes lasted from a few seconds to several minutes, with their frequency gradually increasing from once every few days, to multiple per day. As those progressed, lower limb paresis became persistent. No sensory or pain deficits were described, as well as no bowel and bladder dysfunction, nor the involvement of the upper limbs. The patient was taking trazodone and sertraline, for the treatment of depressive disorder, partially caused by the presence of the aforementioned neurological symptoms.

During neurological examination, strength in lower limbs was measured at MRC grade 3+/5 on the Medical Research Council (MRC) scale in the right limb and MRC grade 4+/5 in the left limb. Bilateral spastic hypertonia of the lower limbs (assessed as grade 3 in Modified Ashworth Scale for Spasticity), with hyperreflexia, was present. Bilateral Babinski and Rossolimo signs were observed, as well as patellar and ankle clonuses. Upper-extremity strength, sensation, and coordination were normal, with no clinical signs suggestive of cervical myeloradiculopathy. The patient’s functional impairment corresponded to Nurick grade V. Romberg’s test was negative, providing no clinical evidence of significant posterior column dysfunction.

**Figure 1:**
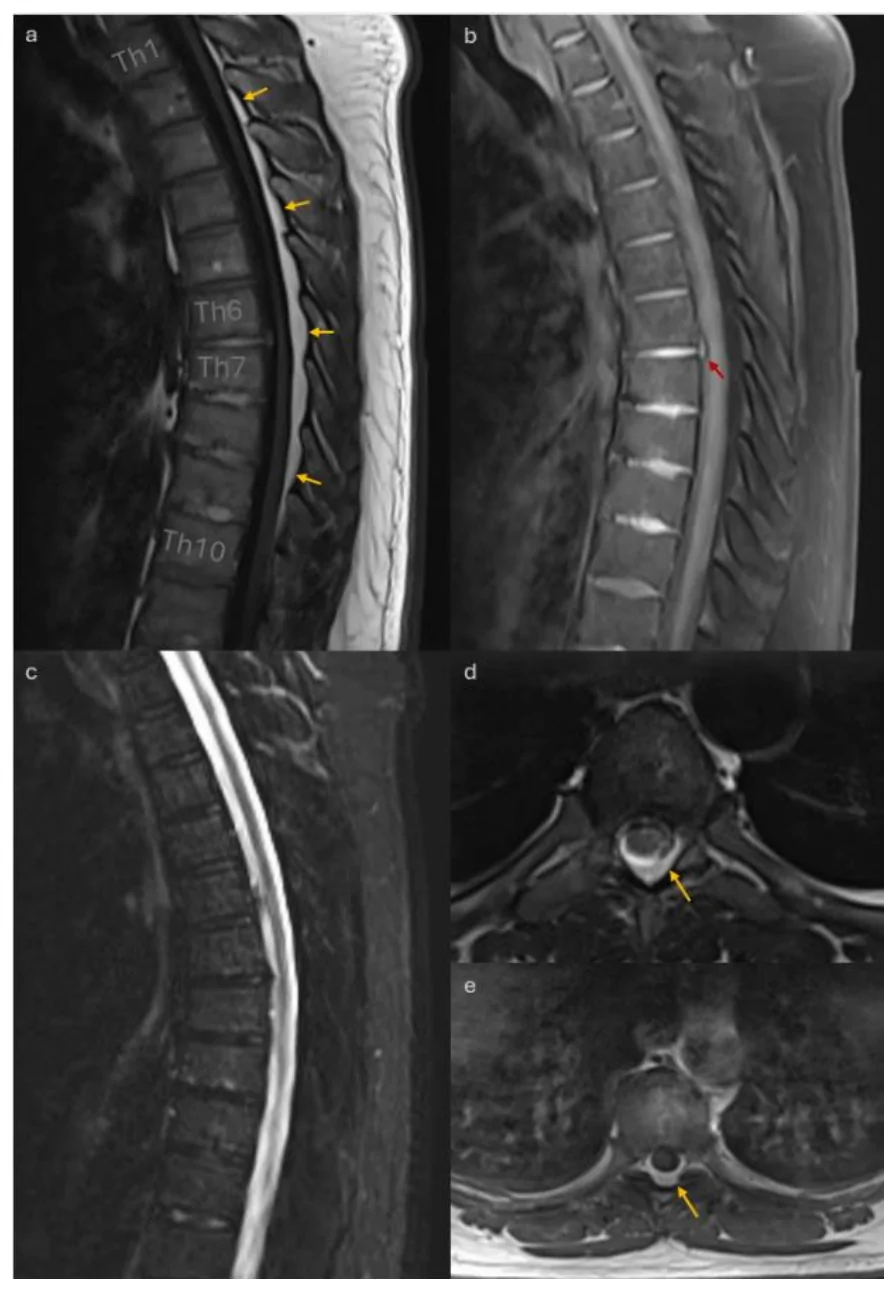
MRI of the thoracic region. Yellow arrows—epidural adipose tissue; Red arrow—Th6/Th7 intravertebral disc herniation. (**a**)—T1WI sequence, sagittal view; (**b**)—T1 Fat Saturation sequence, sagittal view; (**c**)—STIR sequence, sagittal view; (**d**)—T2WI sequence, transverse view, contrast enhancement; (**e**)—T2WI sequence, transverse view.

Under the suspicion of thoracic myelopathy an MRI with and without contrast, of the thoracic spine was performed (Figure 1). Initial radiological reading concluded a presence of Schmorl’s nodes in the vertebral body of Th8–Th10, sporadic osteophytic changes and a Th6–Th7 intervertebral disc herniation, measuring 3 mm in the AP axis. Thecal sac was measured at 9 mm, and no spinal cord signal abnormalities were observed. No stenosis of the vertebral column, or the intervertebral foramina was originally noted.

Further neurological diagnostic workup was carried out. Blood morphology, iron panel, vitamin B12, and folic acid levels were ordered to exclude subacute combined degeneration of spinal cord. Blood smear did not reveal abnormal cells or an increased reticulocyte count. Results showed slightly lower than normal range erythrocyte count (4.22 × 10^12^/L), slightly increased MPV (11.2 fl) and borderline vitamin B12 deficiency—which was corrected without clinical improvement. Biochemical and cytological analysis of cerebrospinal fluid (CSF) were within normal range. Serological testing excluded neuroborreliosis and presence of anti-MOG or anti-AQP4 antibodies, however oligoclonal type V band was discovered in blood serum. Further analysis of the serum immunoglobulins revealed a subtle clonal expansion of lambda IgG, but no hematooncological disorder was diagnosed. No pathologies of peripheral nerves were found by electroneurographic studies or by the ultrasound of the lower limbs, including the piriformis muscle.

The patient was subsequently referred to our facility, where the previously obtained MRI was reassessed. Upon repeat review of the MRI, extensive posterior epidural fat accumulation was identified at the Th2–Th9 levels, causing moderate compression of the thecal sac. The lesion demonstrated signal characteristics consistent with adipose tissue, including high signal intensity on T1-weighted and T2-weighted images with suppression on fat-suppressed sequences, and was provisionally diagnosed as spinal epidural lipomatosis (SEL). At the level of the previously reported Th6–Th7 disc prolapse, the anterior disc pathology and posterior epidural adipose tissue contributed to focal narrowing of the spinal canal. The DuS/EF and EF/SpiC indices were estimated at 1.63 and 37%, respectively. Because the original Borré criteria were developed for the lumbosacral region, these measurements were considered descriptive and were not used to assign a Borré grade. According to the Manjila classification [8], the lesion was classified as type IIb-α, indicating a posterior, moderately severe lesion with an associated degenerative abnormality. The patient did not report any previous spinal imaging studies performed before the onset of his neurological symptoms. Therefore, no prior imaging was available for comparison or for assessment of the temporal development of the epidural adipose tissue.

**Figure 2:**
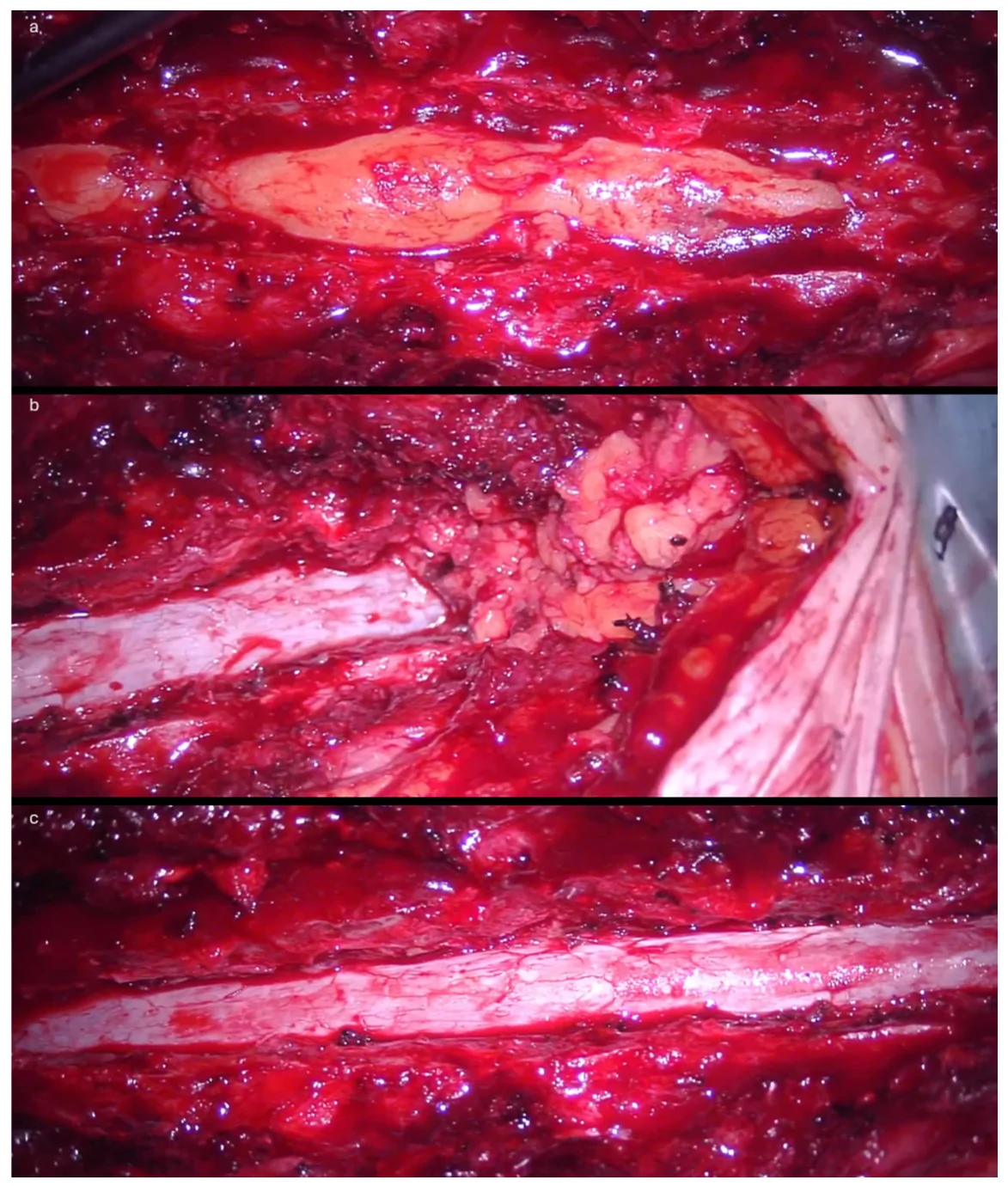
Intraoperative pictures. (**a**), (top)—view seen after laminectomy; (**b**), (middle)—mass of SEL tissue retracted rostrally after dissection from the dura; (**c**), (bottom)—the dura after complete debulking.

The patient was subsequently qualified for surgical decompression. No repeat thoracic MRI was obtained before surgery. During the preoperative period, the patient’s neurological function deteriorated rapidly. Given the progressive neurological deficit and the structural compression already demonstrated on MRI, the decision was made to proceed with surgical decompression without delaying treatment for repeat imaging. Bilateral thoracic laminectomy from Th2 to Th9 was performed to expose the epidural space. The epidural adipose tissue was carefully dissected from the underlying dura and completely resected (Figure 2). Following decompression, the posterior elements were reconstructed using a laminoplasty-assisted technique. Microdiscectomy of the Th6–Th7 intravertebral disc prolapse was not performed. Histopathological examination demonstrated mature adipose tissue without cytological atypia, lipoblasts, necrosis, or other malignant features.

**Figure 3:**
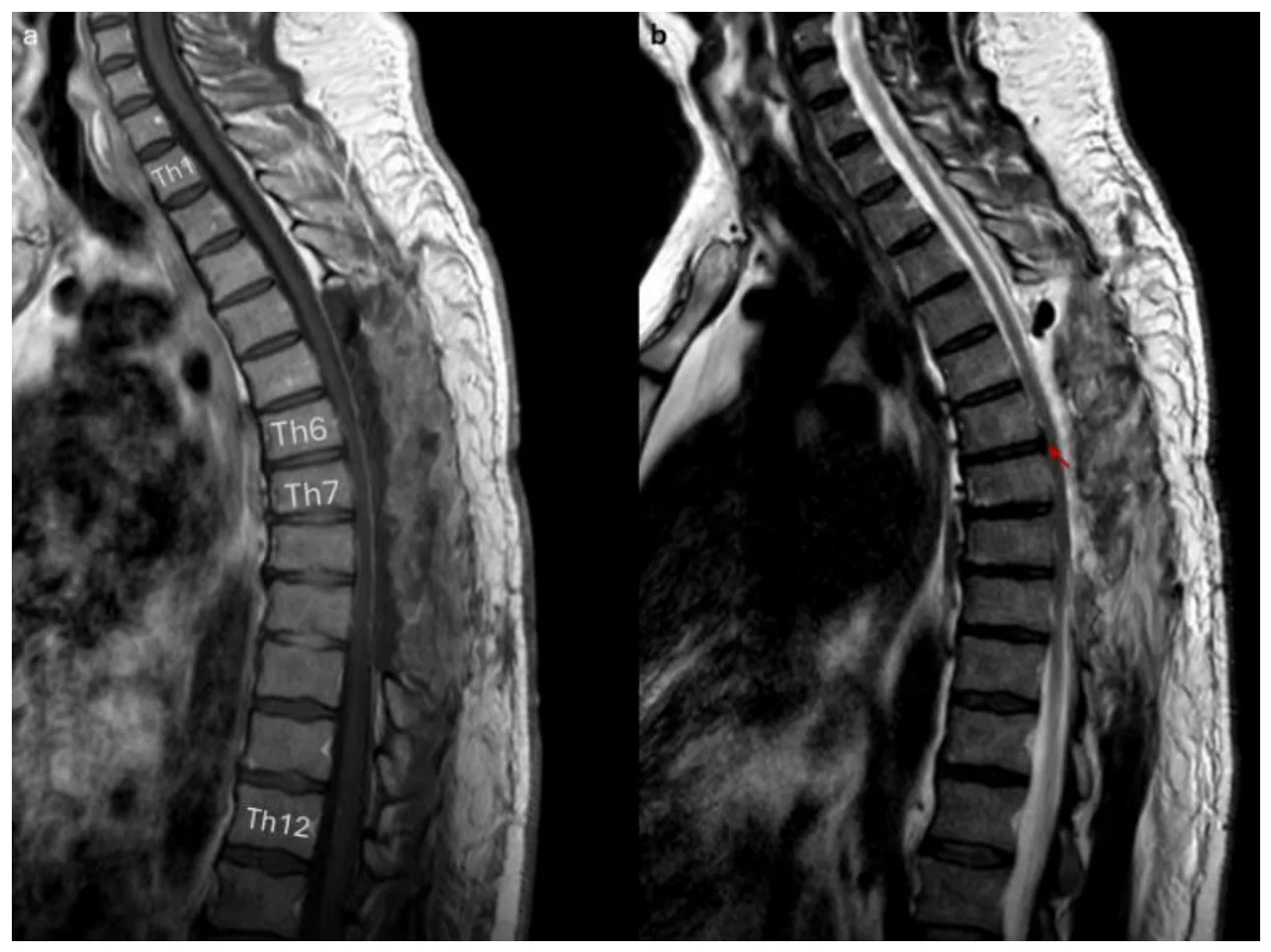
MRI of the thoracic region, post-operatively. Red arrow—Th6/Th7 intravertebral disc herniation. (**a**)—T1WI sequence, sagittal view; (**b**)—T2W1 sequence, sagittal view.

No complications occurred during the surgery or throughout the post-operative period and subsequent imaging showed successful thecal decompression (Figure 3.). Partial improvement of the neurological symptoms was observed immediately after surgery. The patient was discharged, with recommendation of further physical therapy and weight loss (with prescription of an GLP-1 agonist) to prevent SEL re-occurrence. No dedicated CT examination of the chest or abdomen was performed for the purpose of assessing extraspinal lipomatosis. No additional extraspinal lipomatous lesions were identified on the imaging studies available for clinical evaluation.

At 4 months after discharge, lower-extremity strength had improved to MRC grade 5/5 bilaterally, while lower-extremity spasticity had decreased from grade 3 to grade 2 on the Modified Ashworth Scale. Patellar reflexes remained brisk, and ankle reflexes were also brisk, but with bilateral foot clonus. Babinski and Rossolimo signs were no longer present. The patient was subsequently qualified for botulinum toxin treatment to further reduce residual lower-extremity muscle rigidity and continued intensive rehabilitation. Patient’s neurological status allows for long distance physical activity, such as biking and walking for more than 5 km.

At 11 months after surgery, the patient demonstrated sustained neurological improvement, corresponding to Nurick grade II. He was able to undertake prolonged physical activity, including cycling and walking for more than 5 km. A substantial improvement in his mental well-being was also reported. The patient expressed satisfaction with the functional improvement achieved following treatment. Because of the previously identified oligoclonal type V band and subtle clonal expansion of lambda IgG, he remains under clinical surveillance. The patient has given a conscious and written consent to use his medical history, with appropriate imaging and data to report and publish this case.

## 3. Methods

**Figure 4:**
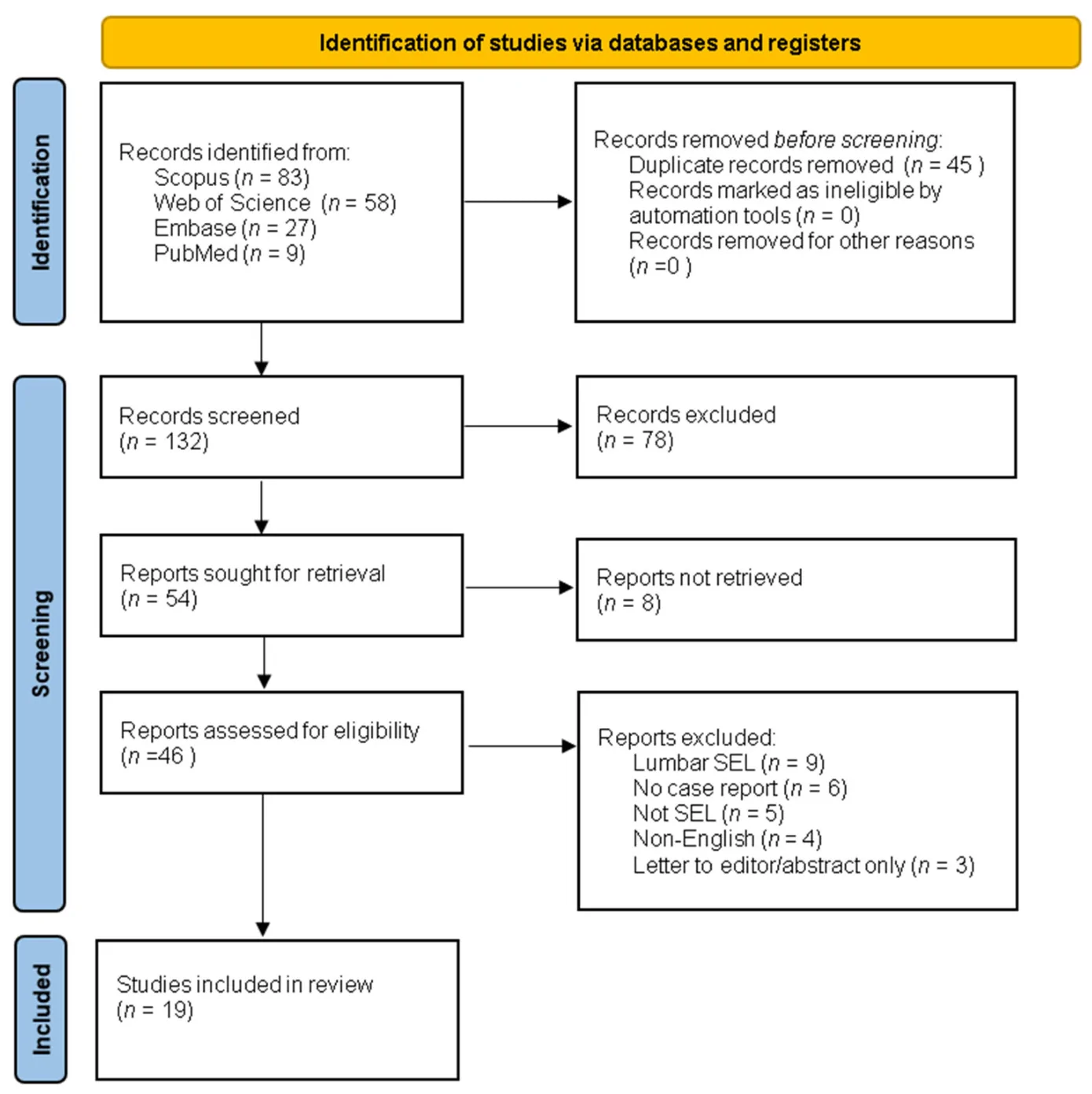
2020 PRISMA flowchart diagram.

We performed a systematic review of case reports of SEL occurring or co-occurring in the thoracic region. The literature was found on PubMed, Scopus, Embase, and Web of Science databases, after using search terms: “(thoracic) AND (“spinal” OR “epidural”) AND (lipomatosis)” and filters: [English], [humans] and [year 2020–2026], with search date of 19 February 2026. Inclusion criteria consisted of peer-reviewed studies, English language and publication between 2020–2026. This review was performed in accordance to the PRISMA (Preferred Reporting Items for Systematic Reviews and Meta-Analyses) guidelines (Figure 4) [9] and has not been registered PROSPERO database. Articles with spinal lipoma, SEL that was not located in the thoracic region, or was located in an unknown region, were excluded. Two reviewers independently evaluated each study, with disagreements resolved by discussion and, when necessary, consultation with a third reviewer. Symptoms were marked as present or absent only if original articles described them—otherwise the Not Available (NA) label was applied. In one case, the extent of SEL lesion was not specifically reported; however, involvement of thoracic and lumbar regions was stated. Radiological measurements of DuS, EF, and SpiC were approximated on sagittal projections in selected cases because complete imaging datasets were not available. Measurements were performed at the level of, and parallel to, the upper endplate of the vertebra corresponding to the most affected segment. Because these measurements did not follow the standardized methodology originally proposed by Borré et al. [7] for lumbosacral SEL, they were not used to assign a formal Borré grade and were considered descriptive only. For selected cases in which the available imaging projections and image quality allowed assessment, the Manjila classification was applied descriptively. This classification uses a 3 × 3 grid on axial MRI images to characterize the severity and anatomical distribution of epidural fat, including posterior/dorsal, anterior/ventral, and circumferential patterns. This method can reliably distinguish the anatomical location of thoracic SEL into dorsal, ventral, and circumferential lesions, which in turn, helps plan approaches for surgical decompression. In the present review, the classification was used to describe the morphology of reported lesions and to facilitate comparison between cases and its application in this review should be considered descriptive rather than a validated assessment of lesion severity.

## 4. Results

We analyzed 21 cases of SEL involving the thoracic region, from 19 articles included in our review. In 12 (57.1%) cases the patients were male, in 9 (42.9%) female. The mean age was 42 years (SD = 20.1 years). Reported etiological associations and comorbidities varied across the included cases. In 7 cases SEL was associated with Type 1 Diabetes Mellitus (T1DM), in 6 cases with iatrogenic causes (prolonged or intrathecal steroid therapy), 4 cases were associated with obesity or metabolic syndrome, 3 patients were diagnosed with Cushing’s syndrome and 1 patient was diagnosed with CLOVES syndrome (PIK3CA gene mutation, causing adipose tissue overgrowth, noted in the Table 1 as an “other” cause). Due to overlap in risk factors 2 patients were marked as having two etiological associations/comorbidities (combination of steroid therapy and T1DM in one case and of steroid therapy and metabolic syndrome in the second one) In the cases where Body Mass Index (BMI) was provided (*n* = 12), most patients were either overweight (*n* = 5) or obese (*n* = 6)—this, however might be a bias, due to low reporting rate of this parameter in individuals with normal body weight. Because these associations were derived from individual case reports and frequently overlapped, they should not be interpreted as independent risk factors or as evidence of causality.

Presentation of SEL lesion location, in relation to the spinal segments, is provided in Table 1. (in one article the segments involved were not directly specified, however the involvement of the thoracic region was stated).

20 (95%) patients presented with motor dysfunction of a moderate grade (which was defined as a report of “paraparesis”, or “weakness”, denoted as 1 in “Motor symptoms” in Table 2) in 10 (47%) patients—or severe grade (which was defined as a report of “paralysis”, “paraplegia”, or proximal extremity strength of =<1/5 on MRC scale, denoted as 2 in “Motor symptoms” in Table 2) also in 10 cases. Sensory deficits were also common and noted in 16 patients (76%). Sphincteric dysfunction was reported in only 8 (38%) cases, while presence of clonus and Babinski signs in 5 (23%) patients each. Muscle stretch reflexes were described as decreased in 4 (19%) patients, while an equal number of articles reported increased reflex strength. One patient had decreased abdominal and cremasteric reflexes, with increased patellar reflexes. In 3 patients’ additional involvement of upper extremity was reported. Since many of the aforementioned symptoms were not specifically mentioned, the actual occurrence might be underreported.

While SEL is typically thought of as a subacute/chronic cause of myelopathy, in select cases symptom onset might be sudden. In 5 cases from our review, the time from appearance of symptoms until presentation was less than a week, with one case report describing SEL as a “stroke mimic”. In the rest of the cases, in which this metric was provided (*n* = 12), the mean time until presentation was 17.7 months, with a median of 8 months, highlighting the variable onset of SEL associated myelopathy.

12 patients with symptomatic thoracic SEL were observed to have co-occurring spinal conditions, with 5 cases reporting degenerative changes.

Management of the SEL was mostly surgical, with 15 (71%) of the patients undergoing a decompressive and/or debulking procedure. 3 additional patients were qualified for surgery, however ultimately declined. Within the surgical group most patients experienced a favorable outcome, with 5 cases noting a modest to moderate improvement and 8 of the patients having a near or complete resolution of symptoms. Outcome of a single surgical patient was deemed unfavorable; however, this is due to a co-occurrence of acute SCI, with his post-operative state being classified as ASIA B score. Three minor complications (lymphorrhea and 2 cases of initial worsening neurological function) and one major (need for a re-operation) were noted. 2 patients were treated conservatively (due to systemic comorbidities making them poor surgical candidates)—while no follow-up information exists for these patients, at the time of hospitalization their function did not improve. In one case, diagnosis of SEL (co-occurring with an epidural abscess) was described, but no information about further treatment of the patient was provided.

## 5. Discussion

Spinal Epidural Lipomatosis is a rare benign overgrowth of adipose tissue, occurring within the epidural space of the vertebral column. Common causes of SEL are corticosteroid therapy, obesity, Cushing syndrome, and metabolic syndrome. Less common patho-etiological factors include inflammatory, vascular, degenerative, and infectious causes. The distribution of reported comorbidities and etiological associations in our review should be interpreted with caution. The relatively frequent reporting of type 1 diabetes mellitus, corticosteroid exposure, obesity/metabolic syndrome, and Cushing syndrome does not indicate their prevalence among patients with thoracic SEL and cannot be used to estimate relative risk or establish causality. The reviewed evidence consisted predominantly of individual case reports and is therefore particularly susceptible to selection and publication bias. Conditions considered clinically relevant to SEL may be preferentially reported, whereas patients without notable comorbidities may be less likely to be described in the literature.

While SEL is often asymptomatic and is detected incidentally [10], patients with this disease might become symptomatic in presence of other pathologies of the vertebral column, or spinal cord [11]. Due to lack of data, scientifically validated guidelines for standards of care for patients with SEL (especially of the thoracic region) do not exist—as such decisions regarding surgical management must often be individualized and be taken on a case-to-case basis. This is especially important in patients with co-occurring spinal disorders and/or acute worsening of the neurological state.

The original MRI criteria for detection and grading of SEL were proposed by Borré et al. in 2003 [5]. These criteria were developed using measurements obtained in the lumbosacral region, and their applicability to thoracic SEL remains uncertain. In particular, the anatomical distribution of epidural fat differs between spinal regions, and a standardized thoracic measurement level is difficult to define. Therefore, Borré-derived measurements in thoracic cases should be interpreted cautiously unless obtained using a validated thoracic methodology. This is also a likely reason why the initial radiological reading of the patients with MRI did not lead to the diagnosis of SEL (combined with the use of T1 Fat Saturation sequence).

The Manjila classification provides an alternative morphological description based on the distribution and severity of epidural fat on axial imaging. In our review, this classification was applied only to cases in which adequate images were available. Although it may facilitate description of dorsal, ventral, and circumferential patterns and may potentially assist surgical planning, its use in thoracic SEL should currently be regarded as descriptive because dedicated validation in thoracic disease is lacking.

We present a case of a patient with symptoms consistent with upper motor neuron syndrome caused by co-occurrence of thoracic SEL and intervertebral disc herniation, which was initially undiagnosed despite prior MRI evaluation. Further diagnostic evaluation caused unnecessary time loss for the patient, which led to disease progression. Despite this, the patient’s condition improved after the operation and months of physical therapy, allowing them to return to daily function—however it is unclear if earlier intervention would allow for full recovery, without the need for botulotoxin injections.

Our case also illustrates the potential synergistic effect of coexisting structural abnormalities. At Th6–Th7, the anterior disc prolapse and posterior epidural adipose tissue produced a focal area of maximal canal narrowing. Although neither abnormality alone was initially considered sufficient to explain the severity of the patient’s neurological syndrome, their combined effect may have contributed substantially to spinal cord compression. Marked neurological improvement following decompression and complete resection of the SEL, without surgical treatment of the disc herniation, supports the interpretation that SEL was the predominant contributor to the patient’s myelopathy. This observation emphasizes the importance of evaluating the spinal canal rather than attributing symptoms to a single radiological abnormality in isolation. This case should not be interpreted as establishing a general diagnostic or therapeutic algorithm. Rather, it highlights the importance of integrating the clinical examination with careful assessment of the entire spinal canal and all coexisting structural abnormalities when determining the likely cause of myelopathy.

A notable part of our patient’s overall health state was the bi-directional relationship between his somatic disease and mental disorder. Exacerbations of depressive symptoms and anxiety were confounding factors triggering episodes of lower limb muscle spasms. Conversely, medications he was prescribed (trazodone and sertraline), could be partially responsible for weight gain [12], leading to further proliferation of SEL.

Our literature review highlights that thoracic SEL often occurs in individuals, which also have other co-existing diseases of the spinal cord or the vertebral column. This could explain why symptomatic thoracic epidural overgrowth seems to be smaller in size than lumbar SEL lesions, as synergistic effect between multiple structural abnormalities might be the reason for the development of myelopathy. Motor symptoms were most common, existing in all but one patient, with sensory deficits being second most common. True rate of sphincter dysfunction, Babiński sign occurrence, development of reflex abnormalities and clonus might be understated, as most authors omitted describing the existence of these symptoms. Surgical operation remains valid for treatment of symptomatic SEL of the thoracic region; however, not enough data exists to make claims about its effectiveness. The long-term risk of reaccumulation of epidural adipose tissue following surgical decompression and resection also remains poorly characterized. Consequently, long-term clinical and radiological follow-up may be appropriate, particularly in patients with persistent obesity or other predisposing metabolic or endocrine conditions. Despite different surgical approaches used by authors of the studies in our review, there does not seem to be a technique that is statistically superior to others. This, however, cannot be proven in such a small sample size of patients. Few authors have demonstrated the utility of less invasive techniques—such as skip laminectomies [13], alternating hemilaminectomies [14,15], or endoscopic procedures [16]—in successfully treating SEL. Those approaches have many theoretical benefits over classical laminectomies and might reduce risk of adverse effects compared to an open approach. While historically conservative management was largely unsuccessful in treating SEL (except those cases where exogenous steroids were responsible for the disease), currently singular cases [7] the use of GLP1-recepor agonists might prove a valuable treatment option in patients who do not qualify for an intervention. These drugs are also used in patients after surgical intervention as a secondary prevention of SEL re-occurrence (such as in our case).

An additional consideration in patients undergoing decompression for clinically significant myelopathy is perioperative spinal cord perfusion. At present, there are no clearly established mean arterial pressure (MAP) targets specifically validated for elective decompressive surgery in patients with chronic non-traumatic compressive myelopathy, including thoracic SEL. Available recommendations regarding MAP augmentation are derived predominantly from studies of acute traumatic spinal cord injury [17] and therefore cannot be directly extrapolated to patients undergoing elective decompression for structural lesions. Nevertheless, avoidance of hypotension and maintenance of adequate spinal cord perfusion are reasonable perioperative considerations, with hemodynamic management individualized according to the patient’s neurological status, comorbidities, and surgical circumstances. Further studies are required to determine whether specific perioperative spinal cord perfusion targets are associated with improved neurological outcomes in non-traumatic compressive myelopathy.

### Differential Diagnosis

**Figure 5:**
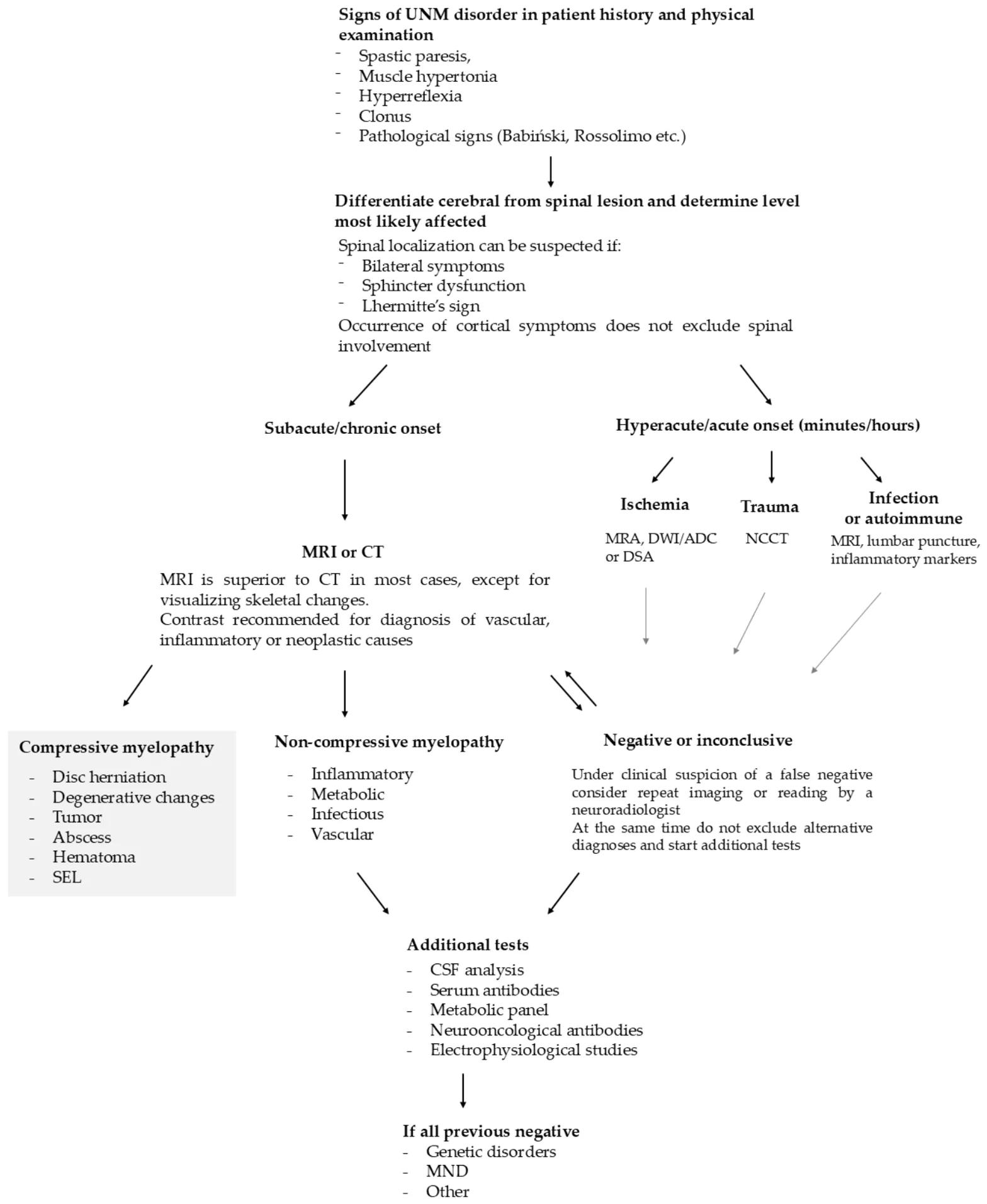
Simplified approach to myelopathy.

While we focus on SEL, the differential diagnosis for subacute lower limb spastic paresis is broad (Table 3, Figure 5), with other possible etiologies including structural changes of the spine, such as degenerative changes, tumors or epidural abscess [18]. A priority in differential diagnosis of myelopathies is exclusion of possibly reversible causes, even when those diseases might be rarer (spinal arteriovenous malformations) than relatively common irreversible etiologies (such as genetic), those causes should be tested for first, which decreases the chance of missed diagnosis for a possibly treatable pathology [19,20,21].

Structural causes remain among the most important considerations in patients presenting with progressive spastic paraparesis [18,22]. Degenerative abnormalities may coexist and produce an additive or synergistic effect on spinal cord compression. In addition to disc herniation and spinal canal stenosis, relevant findings include ligamentum flavum thickening or hypertrophy, facet arthropathy, synovial cysts, spondylolisthesis, and disc-osteophyte complexes. Careful assessment of the entire spinal canal is therefore necessary, particularly when more than one structural abnormality is present.

Vascular causes should also be considered because several spinal vascular disorders are potentially treatable. Spinal cord ischemia, particularly involving the anterior spinal artery territory, may present with an acute motor-predominant syndrome. Spinal epidural or subdural hematoma typically has a sudden onset and may evolve rapidly; an underlying vascular malformation, including a cavernous malformation, should be considered when clinically appropriate [22,23]. Spinal arteriovenous fistulas, particularly dural or perimedullary fistulas, may cause progressive myelopathy through venous hypertension and congestion and can be present as a chronic progressive myelopathy [24]. In selected patients, spinal angiography may therefore be required despite nondiagnostic initial MRI findings [24].

Given the suspicion of thoracic myelopathy, an MRI of the spine should be obtained. This modality allows for detection and differentiation of many common causes of spinal disorders. For instance, the epidural abscess should present itself as a structure with fluid-equivalent signal intensity on T2-weighted images with rim enhancement and a hypointense center [25], while meningioma should form a dural-based mass that is isointense or hypointense to gray matter on T1-weighted images and isointense or hyperintense on proton density and T2-weighted images [26]. Most common vertebral tumors are prostate cancer, breast cancer, lung cancer, or multiple myeloma metastasis, which have variable radiological presentation, but commonly occur as multiple lesions [27]. While reviewing the scans, it is important to actively search for such characteristic findings; however, this might lead to a diagnostic trap, where—while looking for specific signs—subtle structural changes can be missed. This is evident in our example, which shows that even mild EF overgrowth can have a synergistic effect with other spinal pathologies—each of which would not be attributed to the clinical picture of the patient and radiological reading should be approached from a more holistic view.

MRI characteristics for SEL include mainly T1 hyperintense and T2 intermediate/hyperintense structures with noticeable fat suppression. A classical indication of SEL at axial MRI image is the presence of the stellate thecal sac, or the “Y” sign [1,28]—the latter however seems to be more common in the lumbar region, where the epidural fat is both ventrally and dorsally to the thecal sack, unlike within the thoracic region where it is almost exclusively dorsal to the spinal cord (as discussed in literature [29] and shown in our review, as all lesions were Manijla grade II), where the epidural fat is present ventrally and dorsally to the spinal cord, unlike the thoracic region where its mostly located dorsally.

Thus, even if the initial MRI is negative, under enough clinical suspicion of structural cause of myelopathy it is reasonable to obtain a repeat set of imaging (possibly with contrast, specific sequence or another modality) or a second radiological opinion. Signs of upper motor neuron disease, with preserved peripheral nerve studies, negative biochemical serological tests, and no abnormalities in the CSF analysis should always raise suspicion of compressive spinal pathology even when initial imaging is inconclusive.

**Table 3:** Other possible causes of myelopathy.

**Potential etiology**	**Additional information** Despite “characteristic” signs or symptoms, many of diseases described below have overlapping clinical pictures, heterogenous presentation and even patients with the same diagnosis can have different manifestations. This means that additional tests are paramount, and differential diagnosis might remain wide for a long time from symptom onset. Transverse myelitis can be caused by multiple listed disorders, as such it won’t be described separately.
**Structural** [18,19]	Commonly different degenerative changes co-occur with each other, producing an additive effect. CT scan is more useful for skeletal lesions.
Spinal canal stenosis	- most common cause of myelopathy - typically, cervical - reduced spinal mobility
Disc herniation	- typically, lumbar - pain after provocation maneuvers
Tumors and neoplasms	- often asymmetrical symptoms - radicular and localized back pain are common - onset and progression of clinical symptoms are very variable - MRI with contrast is recommended in diagnostic process (variable radiological presentation) - prostate, lung and breast cancers are common causes of vertebral metastasis, causing cord compression
**Vascular** [23,24,30]	Even though rare, vascular etiologies are potentially reversible, as such should be excluded before diagnosing other causes.
Spinal cord ischemia	- most often infarction of anterior spinal artery - hyperacute or acute onset - Diffusion Restriction on MRI is very sensitive in an acute phase
Hematoma	- sudden onset and fast progression, but might have no clinical symptoms - often idiopathic - changing T1 and T2 signals over time (isointense on T1 and iso-/hypointense on T2 in acute stage)
A-V malformations	- MRI with contrast can show characteristic signs (flow voids, missing piece sign), but tis not sensitive enough for definitive exclusion—Digital Subtraction Angiography remains the gold standard
**Autoimmune** [31,32]	Attributing spinal lesions to autoimmune disorders might be a cause of misdiagnosis, due to relatively loose diagnostic criteria.
Neuroautoimmune - MS - MOGAD - NSMOD - Anti-GFAP astrocytopathy	- While symptoms usually appear acutely during attacks, deficits might persist and become chronic - positive Lhermitte’s sign - clinical (vision loss, nausea, etc.) and radiological signs of cerebral involvement - oligoclonal bands or pleocytosis in CSF - MS lesions are usually asymmetrical and extend less than 3 spinal segments, unlike NMOSD lesions which are central and more than 3 segments long - McDonald criteria for MS diagnosis, anti-AQP4 IG antibodies for NMOSD and anti-MOG IG antibodies for MOGAD - Meningoencephalomyelitis (preceded by a viral infection) and good steroid response are hallmark clinical features of anti-GFAP astrocytopathy
Systemic autoimmune	- mostly non-characteristic lesions on MRI (except subdural contrast enhancement and/or trident sign in neurosarcoidosis) - often based on occurrence on presence of the underlying disease and exclusion of other causes of myelopathy
Paraneoplastic syndromes	- can be confirmed by presence of neurooncological antibodies, but negative result cannot exclude the diagnosis - often neurological symptoms precede the threshold for clinical detection of the primary tumor—repeat diagnosis toward neoplasm is reasonable after exclusion of other causes
**Metabolic, toxic and deficiency related** [33]	Might present similarly to hereditary spastic paraplegia, but some are possibly reversible.
Vitamin B12 and copper deficiency	- MRI lesions of the posterolateral part of the spinal cord might be present in both deficiencies; however, these are not very sensitive - megaloblastic/hyperchromatic anemia in vitamin B12 deficiency - history of malabsorption syndromes, nitric oxide exposition, or previous GI tract resection
Hepatic, post-radiotherapy and post-chemotherapy	- diagnosis based largely on exclusion of other causes of myelopathy in patients with appropriate history
**Infectious** [34,35,36]	Usually, having acute presentation, with other signs of inflammation, however, can present chronically and/or without systemic symptoms in immunocompromised patients. Consider the presence of typical risks for HIV and other transmittable diseases.
Retroviral myelopathy - HTLV1 - HIV	- HTLV1 myelopathy (Tropical Spastic Paraparesis; TSP) occurs predominantly in endemic regions and usually has negligible sensory involvement. Diagnosis is based on serology and molecular testing for HTV1 infection and the presence of T-cell lymphoma should also be excluded. - Myelopathy is a late complication of HIV and often coexists with other neurological manifestations of AIDS and co-occurring infectious causes
Other - Syphilis - TB - Herpesvirus - Fungal infection	- Usually, syphilis causes degeneration of the dorsal columns, without spastic paresis, however meningomyelitis can cause such symptoms - TB causes myelopathy in many different mechanisms (myelitis, leptomeningitis, vertebral lesions), as such imaging is variable
**Genetic** [37,38,39]	Should be considered after excluding other causes of UNM disease, or if there is an appropriate family history. This group is highly heterogenic, with many genetic variants, different possible phenotypes, as well as variable symptom onset.
Hereditary spastic paraplegia	- Childhood onset, developmental cognitive impairment, neuropathy, visual loss and opthalmoparesis can suggest HSP, but are nor specific nor characteristic
Leukodystrophy	- Often cognitive, pseudobulbar, cerebellar and peripheral involvement
Adrenomyeloneuropathy	- Besides neurological symptoms, electrolyte abnormalities characteristic for adrenal insufficiency and elevated serum VLCFA can be present
Other - Lysosomal dystrophies - Krabbe’s disease - Metachromatic leukodystrophy - Vanishing White Matter disease - Types of spinocereberall and spastic ataxias	- Many hereditary diseases present with spastic paraparesis as a part of a larger clinical picture, accompanying disorders of other nervous system structures and different organs—thus if suspected, active searching for such signs should be undertaken, before making a final diagnosis
**Motor neuron diseases** [40,41].	MND and myelopathies share many clinical symptoms and differencing between both is difficult
- ALS - PLS	- Symptoms of LMN involvement, such as radiculo/neuropathies, muscle fasciculations/wasting and ENG abnormalities are present in ALS - Gold Coast criteria can help with ALS diagnosis - Early phase ALS can manifest as isolated spinal lesion and should not be confused as myelopathy - PLS is an UMN disorder which usually begins with symptoms of spastic paraparesis and due to lack of specific diagnostic criteria, should only be considered after exclusion of myelopathy

## 6. Limitations

The major limitations of our study are the small number of identified cases, heterogeneous clinical reporting, incomplete availability of imaging, and the retrospective nature of the available evidence. Because neurological symptoms and signs were inconsistently reported, only findings explicitly described in the original publications were coded as present or absent, which may have resulted in underestimation of their true prevalence. Radiological measurements of DuS, EF, and SpiC were estimated in selected cases without access to complete imaging datasets and therefore should be considered illustrative rather than definitive. Similarly, Manjila grading was based on available images and was applied descriptively; the lack of standardized imaging protocols and dedicated validation of this classification for thoracic SEL limits the reliability and generalizability of these assessments. Finally, the heterogeneous surgical techniques and follow-up periods prevented meaningful comparison of treatment strategies.

## 7. Conclusions

Thoracic SEL is a rare but clinically important cause of compressive myelopathy and may occur in association with obesity, endocrinopathies, or exogenous corticosteroid exposure. Although symptoms most commonly develop gradually, acute or rapidly progressive presentations may also occur. MRI remains essential for diagnosis, but thoracic-specific radiological criteria have not been adequately established. The Borré grading system was originally developed for lumbosacral SEL, and its applicability to thoracic disease remains uncertain, while the Manjila classification may provide a useful descriptive framework for the anatomical distribution of epidural fat but requires further validation in thoracic SEL. Early recognition and timely decompression may prevent permanent neurological impairment in symptomatic patients. Careful reassessment of MRI should be considered when the clinical presentation is suggestive of myelopathy despite an initially inconclusive radiological interpretation, particularly when multiple structural abnormalities coexist. Large-volume, multicenter studies with long-term clinicoradiological follow-up would be necessary to understand the natural history and treatment outcomes, and to form evidence-based guidelines for interventions.

## Figures and Tables

**Table 1:** Patient demographics.

Characteristic	Total (*n* = 21)
Basic demographic information	
Total (*n*, %)	21 (100%)
- Male	- 12 (57%)
- Female	- 9 (42%)
Age (*n*, %)	20 (95%)
- Mean	- 42.35
- Median, SD	- 43.5 (SD = 20.14)
BMI (*n*, %)	12 (57%)
- Mean	- 33.33
- Median (SD)	- 31 (SD = 7.22)
Reported etiological associations/comorbidities	
- Diabetes Mellitus Type 1	- 7
- Iatrogenic/Corticosteroids	- 6
- Obesity/Metabolic Syndrome	- 4
- Cushing Syndrome	- 3
- Idiopathic	- 2
- Other	- 1
Co-occurring spinal conditions	
- Degenerative Changes	- 5
- SCI or Spinal Fracture	- 3
- Scoliosis/Kyphosis	- 3
- Syrinx	- 3
- Other	- 2
Radiological metrics	
DuS/EF ratio (*n*, %)	18 (85%)
- Mean	- 0.66
- Median (SD)	- 0.64 (SD = 0.19)
EF/SpiC ratio (*n*, %)	18 (85%)
- Mean	- 60.61
- Median (SD)	- 61 (SD = 6.48)
Manjila type (*n*, %)	13 (61%)
- IIa	- 2
- IIb	- 4
- IIc	- 7
Treatment	
Surgical (*n*, %)	16 (76%)
- Full or near full recovery	- 7
- Mild to moderate improvement	- 8
- No or incomplete recovery	- 1
- Complications	- 4
Declined surgery (*n*, %)	3 (14%)
Conservative (*n*, %)	2 (9.5%)
- No or incomplete recovery	- 2
NA (*n*, %)	1 (4.7%)

**Table 2:** Legend: The graph at the left side of the table shows the spinal segments with SEL. Sex: [F—Female, M—Male]; S.C.—(co-occurring) Spinal Conditions [1—degenerative changes, 2—SCI or spinal fracture, 3—scoliosis/kyphosis, 4—syrinx, 5—other]; C—comorbidities/etiological associations [0—idiopathic, 1—iatrogenic, 2—obesity/metabolic syndrome related, 3—type 1 diabetes mellitus, 4—Cushing syndrome, 5—other]; PT—Time since symptom onset [m—months; d—days]; Mo—Motor symptoms [1—paresis, 2—plegia], Se—Sensory symptoms [1—incomplete loss, 2—major/complete loss], Pa—Pain [0—did not occur, 1—occurred], Sph—Sphincteric symptoms [0—did not occur, 1—occurred]; Bs—Babiński sign [0—did not occur, 1—occurred]; R—Reflexes [0—normal, 1—reduced, 2—increased, 3—absent], CI—Clonus [0—did not occur, 1—occurred], Co—complications [0—did not occur, 1—occurred]; F—Followup [m—months; d—days]; Mg—Manjila grade. Suffixes: B—bilaterally, R—right sided, L—left sided; NA—Not Available.

C							Th									L			S	#	Author	Year	Sex	Age	BMI	SC	C	PT	Mo	Se	Pa	Sph	Bs	R	Cl	Intervention Type	Co	Outcome	F	DuS/EF	EF/SC	MG
																				1	Manasi Arora	2020	F	48	28	4	3	NA	2B	1B	NA	1	NA	NA	NA	T2–T7 laminoplasty	0	near resolution of sphincteric symptoms, persistant parasthesia and pain	18 m	0.74	57	NA
																				2	Dmytro Ishchenko	2024	F	6	NA	3	5	5 d	2B	1B	1	0	1B	1B	NA	T3–T5 laminoplasty, T2 laminectomy, debulking	1	near full or full recovery	3 m	NA	NA	IIc
																				3	Itay Lotan	2020	F	48	NA	no	3	years	2B	1B	NA	1	NA	NA	NA	conservative	-	negligible improvement	NA	1.09	48	NA
																				4	Itay Lotan	2020	F	43	NA	no	3	36 m	1B	1B	NA	NA	1B	1B	NA	declined	-	-	-	1.21	45	IIa
																				5	Itay Lotan	2020	M	44	NA	no	3	36 m	1B	1B	NA	1	1B	3B	1B	C7–T10 laminectomy, debulking	1	initial worsening in LE strength	3 m	NA	NA	NA
																				6	Matthew T. Neal	2021	M	72	NA	5	0	5 m	2B	1B	1	1	1B	NA	1B	alternating T2–T7 hemilaminectomies, debulking	0	modest strength improvement in LE	6 m	0.60	63	IIc
																				7	Malak El Ayssami	2025	M	37	32	1	2	1 m	2B	1B	NA	NA	NA	2 + 3B	1B	laminectomy, debulking	0	mild ataxic gait, no sphincertic symptoms	3 m	0.52	66	IIb
																				8	Anthony M. Alvarado	2023	F	46	28	no	1, 3	18 m	0	1B	NA	1	NA	1L	1L	T2–T7 open-door laminoplasty, debulking	0	near full or full recovery	3m	0,62	62	IIc
																				9	Luke Mugge	2022	M	72	35	2	1, 2	1 d	2B	NA	NA	1	NA	0	0	T2–T9 laminectomy, debulking	0	ASIA B score, with 0/5 in LE	no	0.68	59	IIc
																				10	Shibani Mehra	2020	M	3	NA	5	1	3 m	1B	NA	NA	NA	NA	NA	NA	NA	-	-	-	0.38	72	NA
																				11	Lukasz Przepiorka	2023	F	51	NA	4	3	72 m	2B	1B	NA	NA	NA	NA	NA	T4 and T10 vertebroplasty, T2–T10 laminoplasty, debulking. 2nd surgery: duroplasty	1	able to walk, persistant parasthesia	12 m	0.65	61	IIb
																				12	Prakash Ghogale	2025	M	56	NA	no	1	3 d	1B	2	NA	NA	NA	NA	NA	conservative (prednisone tapering)	-	fluctuating LE strengh	NA	0.65	61	IIa
																				13	Jonas Salna IV	2023	M	NA	40	1	2	3 d	1R	0	1	NA	NA	NA	NA	declined	-	-	-	NA	NA	NA
																				14	Kshitij Chaudhary	2020	M	33	NA	1, 2, 3	4	0.5 m	1B	1B	NA	NA	NA	NA	NA	T4–T8 laminoplasty, debulking, pituitary microadenoma resection	0	near full or full recovery	6 m	0.79	56	IIc
																				15	Maroua H. B. Salah	2025	M	81	29	2, 5	2	years	1B	NA	NA	NA	NA	NA	NA	declined	-	-	-	0.58	63	NA
																				16	Abdalhai Alshoubi	2023	F	34	46	no	1	1 d	1L	0	1	0	NA	NA	NA	T3–T9 skipped laminectomy	0	near full or full recovery	6 d	0.58	63	NA
																				17	Jaime L. Martí nez-Santos	2020	F	31	24	1, 4, 5	3	1 m	1B	2B	NA	NA	1B	3B	NA	T2–T9 alternating hemilaminotomies, debulking	0	near full or full recovery	12 m	0.49	67	IIb
																				18	Ayman A. Salman	2024	M	17	25	3, 5	0	9 m	2B	1B	NA	1	NA	NA	NA	T6–T10 transpedicular fixation, debulking	0	near full or full recovery	24 m	0.63	61	IIc
																				19	Noha Mukhtar	2022	M	29	37	no	4	24 m	2B	1B	NA	NA	NA	3B	NA	T2–T12 laminectomy and T3–T7 transpedicle fixation, debulking, pituitary macroadenoma resection	0	near full or full recovery	7 m	0.72	58	IIc
																				20	Angelica M. Fuentes	2022	F	63	30	no	1	months	2B	1B	NA	1	NA	3B	1L	T3–L5 alternating hemilaminectomies	1	considerable improvement in sphincertic symptoms	1 m	0.69	59	IIb
		NA																		21	Micheal Roberto	2020	M	33	46	1	4	7 m	1B	NA	1	NA	NA	2B	1	T4–L3 laminectomy, no debulking	0	Back pain rated 2/10, ODI score improved to 26%	4 m	0.43	70	NA

## Data Availability

The original contributions presented in this study are included in the article/Supplementary Material. Further inquiries can be directed to the corresponding authors.

## Supplementary Materials

The following supporting information can be downloaded at: https://www.mdpi.com/article/10.3390/medsci14050559/s1, Table S1: SEL JBI quality assessment, Table S2: EJMCR-CARE Checklist.
