# Supplementary material for: Progressive Spastic Paraparesis Caused by Thoracic Spinal Epidural Lipomatosis: A Case Report and Systematic Review
Source: Med Sci (Basel). 2026 Sep 10;14(5):559. doi: 10.3390/medsci14050559 (PMC13609061; doi:10.3390/medsci14050559)
Supplement: Supplementary file 1 [file medsci-14-00559-s001.zip › Table S2. EJMCR-CARE Checklist.pdf]

EJMCR-CARE Checklist Thoracic Epidural Lipomatosis
CARE Checklist –: European Journal of Medical Case Reports

Item	Topic	Checklist item description	Line/page
1	**Title**	The words "case report" should be in the title along with the area of focus	p.1
2	**Keywords**	Four to seven key words—include "case report" as one of the key words	p.2
3a	**Abstract**	**Background:** What does this case report add to the medical literature?	p.1
3b		**Case Presentation:** chief complaint, diagnoses, interventions, and outcomes	p.1
3c		**Conclusion:** What is the main "take-away" message from this case?	p.1
4	**Introduction/background**	The current standard of care and contributions of this case—with references (1-2 paragraphs)	N/A
5	**Timeline**	Information from this case report organized into a timeline (table or figure)	N/A
6a	**Patient Information**	De-identified demographic and other patient or client specific information	p.2
6b		Chief complaint—what prompted this visit?	p.2-3
6c		Relevant history including past interventions and outcomes	p.2-3
7	**Physical Exam**	Relevant physical examination findings	p.3
8a	**Diagnostic**	Evaluations such as surveys, laboratory testing, imaging, etc.	p.3-7
8b	**Assessment**	Diagnostic reasoning including other diagnoses considered and challenges	p.3-7
8c		Consider tables or figures linking assessment, diagnoses and interventions	p.3-7
8d		Prognostic characteristics where applicable	N/A
9a	**Interventions**	Types such as life-style recommendations, treatments, medications, surgery	p.4-7
9b		Intervention administration such as dosage, frequency and duration	p.4-7
9c		Note changes in intervention with explanation	p.4-6

9d		Other concurrent interventions	N/A
10a	**Follow-up and**	Clinician assessment (and patient or client assessed outcomes when appropriate)	p.6-7
10b	**Outcomes**	Important follow-up diagnostic evaluations	p.6-7
10c		Assessment of intervention adherence and tolerability, including adverse events	p.7
11a	**Discussion**	Strengths and limitations in your approach to this case	p.14-15, 21
11b		Specify how this case report informs practice or Clinical Practice Guidelines (CPG)	p.14-15
11c		How does this case report suggest a testable hypothesis?	N/A
11d		Conclusions and rationale	p.14-15, 21
12	**Patient Perspective**	When appropriate include the assessment of the patient or client on this episode of care	p.7
13	**Informed Consent**	Informed consent from the person who is the subject of this case report	p.7
14	**Additional Information**	Acknowledgement section; Competing Interests (Conflict of Interests statement); IRB approval, (Ethical Committee Approval) when required	N/A

Summary of the case

1	**Patient (gender, age)**	Male, 54 years
2	**Final Diagnosis**	Spinal Epidural Lipomatosis (thoracic region); intravertebral disc herniation (Th6-Th7); Major Depressive Disorder;
3	**Symptoms**	Progressive Spastic Paraparesis
4	**Medications**	GLP1-R agonist, trazodone, sertraline
5	**Clinical Procedure**	Bilateral laminectomy
6	**Specialty**	Neurosurgery, Neurology, Psychiatry, Physical Medicine & Rehabilitation

EJMCR-CARE Checklist Spinal Epidural Lipomatosis
CARE Checklist –: European Journal of Medical Case Reports

Item	Topic	Checklist item description	Line/page
1	**Title**	The words “case report” should be in the title along with the area of focus	
2	**Keywords**	Four to seven key words—include “case report” as one of the key words	
3a	**Abstract**	**Background:** What does this case report add to the medical literature?	
3b		**Case Presentation:** chief complaint, diagnoses, interventions, and outcomes	
3c		**Conclusion:** What is the main “take-away” message from this case?	
4	**Introduction/background**	The current standard of care and contributions of this case—with references (1-2 paragraphs)	
5	**Timeline**	Information from this case report organized into a timeline (table or figure)	
6a	**Patient Information**	De-identified demographic and other patient or client specific information	
6b		Chief complaint—what prompted this visit?	
6c		Relevant history including past interventions and outcomes	
7	**Physical Exam**	Relevant physical examination findings	
8a	**Diagnostic**	Evaluations such as surveys, laboratory testing, imaging, etc.	
8b	**Assessment**	Diagnostic reasoning including other diagnoses considered and challenges	
8c		Consider tables or figures linking assessment, diagnoses and interventions	
8d		Prognostic characteristics where applicable	
9a	**Interventions**	Types such as life-style recommendations, treatments, medications, surgery	
9b		Intervention administration such as dosage, frequency and duration	
9c		Note changes in intervention with explanation	
9d		Other concurrent interventions	

10a	**Follow-up and**	Clinician assessment (and patient or client assessed outcomes when appropriate)	
10b	**Outcomes**	Important follow-up diagnostic evaluations	
10c		Assessment of intervention adherence and tolerability, including adverse events	
11a	**Discussion**	Strengths and limitations in your approach to this case	
11b		Specify how this case report informs practice or Clinical Practice Guidelines (CPG)	
11c		How does this case report suggest a testable hypothesis?	
11d		Conclusions and rationale	
12	**Patient Perspective**	When appropriate include the assessment of the patient or client on this episode of care	
13	**Informed Consent**	Informed consent from the person who is the subject of this case report	
14	**Additional Information**	Acknowledgement section; Competing Interests (Conflict of Interests statement); IRB approval, (Ethical Committee Approval) when required	

Summary of the case

1	**Patient (gender, age)**	
2	**Final Diagnosis**	
3	**Symptoms**	
4	**Medications**	
5	**Clinical Procedure**	
6	**Specialty**	
